# Two vs. three weeks of treatment with amoxicillin-clavulanate for stabilized community-acquired complicated parapneumonic effusions. A preliminary non-inferiority, double-blind, randomized, controlled trial

**DOI:** 10.1515/pp-2019-0027

**Published:** 2020-02-26

**Authors:** José M. Porcel, Lucia Ferreiro, Laura Rumi, Esther Espino-Paisán, Carmen Civit, Marina Pardina, Juan Antonio Schoenenberger-Arnaiz, Luis Valdés, Silvia Bielsa

**Affiliations:** Pleural Medicine Unit, Department of Internal Medicine, Arnau de Vilanova University Hospital, IRBLleida, Lleida, Spain; Department of Pulmonology, University Clinical Hospital of Santiago, Health Research Institute of Santiago de Compostela (IDIS), A Coruña, Spain; Department of Pharmacy, Arnau de Vilanova University Hospital, IRBLleida, Lleida, Spain; Department of Pharmacy, Hospital de Barbanza, A Coruña, Spain; Department of Radiology, Arnau de Vilanova University Hospital, IRBLleida, Lleida, Spain

**Keywords:** amoxicillin-clavulanate, community-acquired pneumonia, empyema, parapneumonic effusion, pleural effusion

## Abstract

**Background:**

The optimal duration of antibiotic treatment for complicated parapneumonic effusions (CPPEs) has not been properly defined. Our aim was to compare the efficacy of amoxicillin-clavulanate for 2 vs. 3 weeks in patients with CPPE (i.e. those which required chest tube drainage).

**Methods:**

In this non-inferiority, randomized, double-blind, controlled trial, patients with community-acquired CPPE were recruited from two centers in Spain and, after having obtained clinical stability following 2 weeks of amoxicillin-clavulanate, they were randomly assigned to placebo or antibiotic for an additional week. The primary objective was clinical success, tested for a non-inferiority margin of<10%. Secondary outcomes were the proportion of residual pleural thickening of>10 mm at 3 months, and adverse events. The study was registered with EudraCT, number 2014-003137-25. We originally planned to randomly assign 284 patients.

**Results:**

After recruiting 55 patients, the study was terminated early owing to slow enrolment. A total of 25 patients were assigned to 2 weeks and 30 patients to 3 weeks of amoxicillin-clavulanate. Clinical success occurred in the 25 (100%) patients treated for 2 weeks and 29 (97%) treated for 3 weeks (difference 3%, 95% CI −3 to 9.7%). Respective between-group differences in the rate of residual pleural thickening (−12%, 95%CI −39 to 14%) and adverse events (−7%, 95%CI −16 to 2%) did not reach statistical significance.

**Conclusions:**

In this small series of selected adult patients with community-acquired CPPE, amoxicillin-clavulanate treatment could be safely discontinued by day 14 if clinical stability was obtained.

## Introduction

Approximately 20% of hospitalized patients with community-acquired pneumonia (CAP) have accompanying pleural effusions on chest radiographs, of which about one-third are categorized as complicated parapneumonic effusions (CPPEs) (i.e. pleural space drainage is required for cure) [1]. Recommended empiric regimens for hospitalized non-intensive care unit (ICU) patients with CAP include a β-lactam agent plus a macrolide (or β-lactam plus a respiratory fluoroquinolone) [2]. However, it has been consistently demonstrated that bacteriology of pleural infections is distinct from that of CAP [3]. In a recent systematic review, Viridans streptococci, part of the normal flora of the mouth and rarely associated with pneumonia, were the most common bacteria cultured from 1097 community-acquired pleural infections (32%), followed by pneumococci (22%), *Staphylococcus aureus* (18.5%) and anaerobes (17.8%) [4]. Moreover, atypical microorganisms, which frequently cause CAP, are very rarely detected in pleural fluid samples by advanced molecular techniques [5]. Consequently, empirical antibiotics chosen for community-acquired pleural infections usually consist of monotherapy with a β-lactam with activity against anaerobes (e.g. amoxicillin-clavulanate), or a combination of β-lactam (e.g. ceftriaxone) and clindamycin [6].

About 5–7 days of antibiotics are considered effective enough for most CAP [7], though more than 70% of patients hospitalized with CAP eventually receive antibiotic treatment exceeding this recommended duration [8]. Conversely, the optimal duration of therapy for CPPE is unknown. Most experts suggest a prolonged course of 3 or more weeks of antibiotics [9]. Given the lack of evidence available to guide the duration of treatment in CPPE this randomized trial was designed to test, for the first time, whether 2  weeks is non-inferior to 3 weeks.

## Materials and methods

### Study design and patients

The ODAPE (Optimal Duration of Antibiotics in Parapneumonic Effusions; https://www.clinicaltrialsregister.eu/ctr-search/trial/2014-003137-25/ES) was a randomized, double-blind, non-inferiority trial of 2 vs. 3 weeks of treatment with amoxicillin-clavulanate in adults with CPPE or empyemas. Initially, six centers pledged to participate in the trial. However, four failed to enroll patients two years after the trial started and were excluded from the study, which was finally conducted at the remaining two academic centers in Spain (Arnau de Vilanova University Hospital in Lleida, and University Clinical Hospital of Santiago in Santiago de Compostela) between March 2015 and March 2019. It was approved by the Ethics Committee of each participating center (CEIC No. 1384), and all patients included in the study provided written informed consent.

CPPE referred to those exudative effusions associated with pneumonia which required chest tube drainage for resolution. Empyema denoted pus within the pleural space. Decisions on whether to insert a chest tube were based on the presence of any of the following conditions: large (about half or more of the hemithorax) or loculated effusions, purulent fluid, pleural fluid pH ≤7.20 (or alternatively glucose<60 mg/dL), or microorganisms on pleural fluid Gram stains or cultures. Eligible patients were those aged 18 years or older with CAP and an associated pleural effusion which met the criteria for being classified as complicated as detailed above. Exclusion criteria included pregnancy, allergy to amoxicillin-clavulanate, active tuberculosis, hospital or health-care associated pneumonia, immunosuppression (e.g. human immunodeficiency virus infection, transplantation, cancer under active oncologic therapy, use of corticosteroids or immunosuppressive drugs), non-parapneumonic empyema (e.g. trauma, thoracic or abdominal surgery), life expectancy of less than 3 months, or isolation of bacteria resistant to amoxicillin-clavulanate.

Patients with CPPE were hospitalized in a non-ICU setting and empirically treated with 2 g/200 mg intravenous amoxicillin-clavulanate every 8 h, which was switched to an oral route (tablets 875/125 mg/8 h) based on clinical improvement (e.g. no fever for at least 24 h, normal vital signs, falling of serum C-reactive protein [CRP]) and feasibility of oral intake. Use of non-steroidal anti-inflammatory drugs (NSAID) and/or oral azithromycin for a maximum of 5 days in combination with the β-lactam, as well as time of hospital discharge were left to the discretion of the attending physician. Finally, intrapleural urokinase (100,000 U every 24 h for a maximum of 6 days) was routinely used in the participating centers for loculated parapneumonic effusions and empyemas.

### Randomization and treatment

At 14 days of β-lactam therapy, counting from the first day of its administration, patients who had achieved clinical stability were randomized to receive placebo vs. amoxicillin-clavulanate (powder for suspension 875/125 mg/8 h) given orally for an additional week (intervention period). Clinical stability at the time of randomization was defined by all of the following: 1) temperature<37.80 °C, resting pulse and respiratory rates lower than 100/min and 24/min, respectively, and systolic blood pressure>90 mmHg, for the last 48 h, 2) chest drain already removed, 3) pleural effusion occupying less than 20% of the hemithorax on a chest radiograph, and 4) reduction of>50% of the serum CRP concentration at diagnosis.

Randomization was accomplished via a central, web-based system (http://randomizer.org) using a computer-generated list of random numbers in a 1:1 ratio, with permuted blocking and stratification by center. The randomization list was prepared and kept at the Pharmacy Service of the Arnau de Vilanova University Hospital which, along with the Pharmacy Service of the University Clinical Hospital of Santiago, dispensed the allocated treatment. Antibiotics and placebo were delivered in non-labeled identical envelopes in powder form to be taken orally. An external pharmaceutical laboratory (Defabar^®^, Desarrollos Farmaceúticos Bajo Aragón, SL) prepared the placebo with the same taste, color and appearance as the antibiotic. Patients and clinicians were blinded to treatment assignment. At the end of treatment, patients were instructed to return the empty envelopes to the Pharmacy Services to ensure adherence. Researchers evaluated outcomes, which were based on objective clinical, radiological and analytical data. External monitoring of the study was done by SCTFarma from the Biomedical Research Institute of Lleida (IRBLleida, http://www.irblleida.org/en/technical-scientific-services/pharma/).

### Outcomes

The primary end-point was the rate of clinical success, defined as complete or almost complete resolution of symptoms (chest pain, dyspnea) and signs (fever, serum CRP) at 1 week from randomization, with no recurrence of clinical manifestations of pleural infection during follow-up. Conversely, persistent or recurrent symptoms and/or signs of infection, need for further pleural procedures or thoracic surgery, or death attributable to sepsis were designated as clinical failure, provided they were related to the original pleuropulmonary infection. Secondary outcomes, also assessed for non-inferiority, were the lack of residual pleural thickening of>10 mm on a posteroanterior chest radiographic view after 12 weeks from the initiation of antibiotic therapy, and the safety of a longer course of antibiotics.

**Figure 1:**
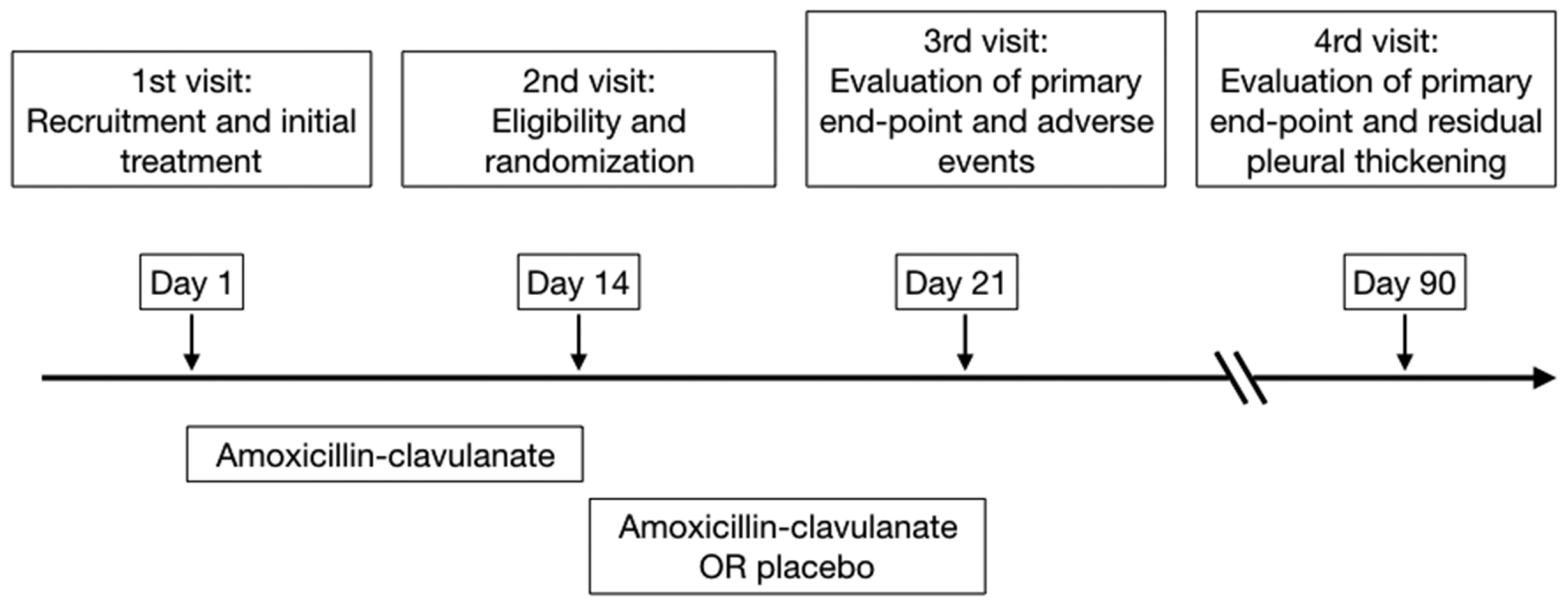
Trial protocol.

Monitoring visits took place at days 1 (eligibility), 14 (randomization), 21 (end of intervention period; primary end-point and adverse events), and 90 (primary end-point and residual pleural thickening) from the initiation of antibiotics (Figure 1). They included evaluation of: inclusion and exclusion criteria, demographics and comorbidities, respiratory symptoms (i.e. chest pain, cough, and dyspnea using the Modified Medical Research Council [mMRC] scale), vital signs, echographic pattern of the pleural fluid at diagnosis (simple or complex), chest radiograph (effusion laterality, pleural loculations, lung consolidation, percentage of the hemithorax area occupied by the pleural opacity using image J software [available at: https://imagej.nih.gov/ij/], residual pleural thickening), biochemical and microbiological pleural fluid data, serum CRP and leukocyte count, blood cultures, prognostic RAPID (renal, age, purulence, infection source, and dietary factors) score [10] at diagnosis, size of chest tubes, duration of chest tube drainage, doses of intrapleural urokinase employed, use of NSAID and antibiotics other than amoxicillin-clavulanate (e.g. macrolides), length of hospital stay, transfer to ICU, referral for surgery, adverse events during the study period, rehospitalizations, and deaths related to pleuropulmonary infection.

### Statistical analysis

The study was designed to have 80% statistical power (two-sided alpha of 0.05) with a non-inferiority margin of 10% between the short and extended antibiotic regimen branches. This non-inferiority boundary is the most widely accepted in similar studies of comparative antibiotic duration [11]. For a primary outcome event of 90% [6], it was estimated that 284 patients would need to undergo randomization. However, the enrollment rate was slower than expected as a consequence of null recruitment in four of the initially participating centers and strict eligibility criteria. Consequently, the study was prematurely terminated, blinded to the clinical outcomes; a circumstance which was communicated and accepted by the local ethics and research committee (CEIC) on May 4, 2019. We calculated 95% confidence intervals (CI) of the difference between the clinical success of the 2- vs. 3-week groups. The non-inferiority hypothesis was considered proven if the 95% CI upper bound was less than 10% points. Mann-Whitney and Fisher’s exact tests were used for numerical and categorical data, respectively. All analyses were conducted using SPSS 22.0 statistical software (Chicago, IL, USA).

## Results

**Figure 2:**
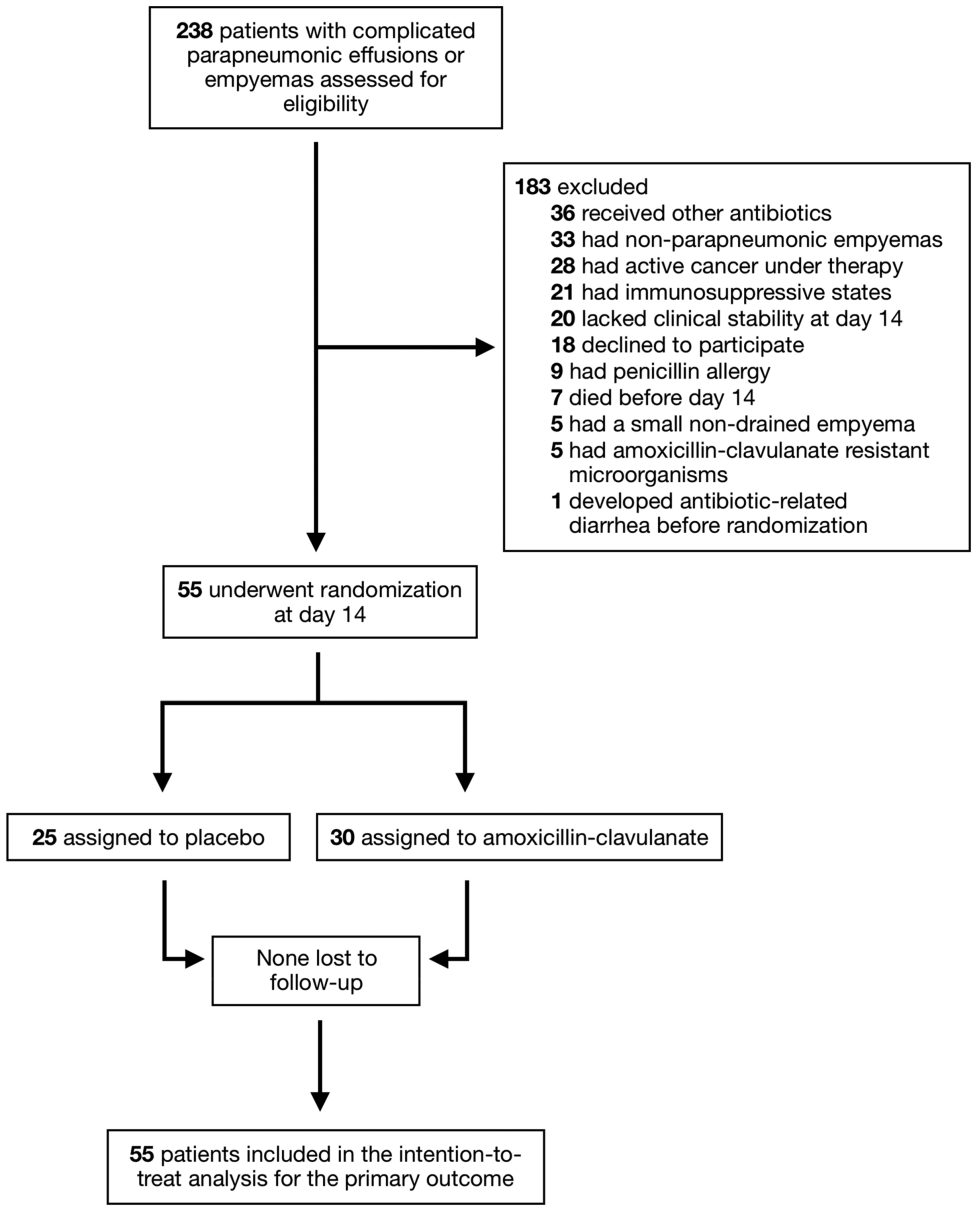
Study flow diagram.

Overall, 238 patients were recruited during the study period, but only 55 (23%) were eligible for enrollment on day 14 following antibiotic therapy (Figure 2). Among the 183 patients who met the exclusion criteria, the most common reasons for non-eligibility were the prescription of antibiotics other than amoxicillin-clavulanate (20%), the non-parapneumonic cause (e.g. post-surgery) of the pleural infection (18%), the presence of cancer (15%) or other immunosuppressive states (11.5%), and the lack of clinical stability at randomization (11%). Of the 55 patients who underwent randomization, 25 were allocated to the 2-week antibiotic treatment regimen and 30 to the 3-week regimen.

Baseline characteristics between study groups were balanced during the study period (Table 1) and, specifically, at the time of randomization (Table 2). Overall, the 55 patients included in this trial had effusions which were predominantly moderate to large in size (41% of the hemithorax occupied by the effusion), loculated (82%), sonographically complex (98%), visibly non-purulent (76%) and culture-negative (67%). A total of 18 microorganisms were isolated from their pleural fluids, including 11 Viridans streptococci*,* 2 *Streptococcus pneumoniae*, and one each of *Streptococcus pyogenes*, *S. aureus*, *S. cohnii*, *Pasteurella multocida*, and *Parvimonas micra*. Patients were treated a median of 5 days (interquartile range [IQR] 4–7) with intravenous antibiotics, and received small (80%) chest catheters that remained in place for 2 (1–4) days, along with intrapleural urokinase (96%), NSAID (75%) and, less often, a concomitant course of oral azithromycin (36%). At the time of randomization, patients were afebrile (median temperature 36 °C), had normal vital signs (median of 80 beats/min, 16 breaths/min and systolic blood pressure 130 mmHg), normal blood leukocyte counts (9330/µL, IQR 7290–10,550/µL), slightly elevated serum CRP (30 mg/L, IQR 13–75 mg/L), and minimal pleural opacities on chest radiographs (11% of the hemithorax, IQR 7–14%). At the end of the intervention period (day 21), vital signs remained stable while other preceding variables further improved or normalized (Table 3).

**Table 1:** Baseline characteristics of the study population.

	2-week treatment group (n=25)	3-week treatment group (n=30)	p-Value
Age, years	60 (50–75)	58 (49–67)	0.20
Male	19 (76)	25 (83)	0.50
Never-smoker	9 (36)	10 (33)	0.98
Comorbidities			
Diabetes	9 (36)	7 (23)	0.30
Heart failure	2 (8)	0 (0)	0.11
COPD	5 (20)	3 (10)	0.29
Chronic kidney disease	3 (12)	1 (3)	0.22
Clinical manifestations at diagnosis			
Temperature, °C	38 (36.9–38.6)	37.9 (36.8–38.6)	1
Chest pain, VAS	8 (6.5–10)	7.5 (6–9)	0.09
Cough	14 (56)	25 (83)	0.03
Dyspnea, mMRC			
0	4 (16)	2 (7)	
1	1 (4)	6 (20)	
2	5 (20)	7 (23)	0.39
3	7 (28)	7 (23)	
4	8 (32)	8 (27)	
Chest radiograph at diagnosis			
Effusion’s size, % of hemithorax	41 (27–56)	44 (36–58)	0.51
Right-sided effusion	15 (60)	10 (33)	0.05
Pleural loculations	20 (80)	25 (83)	0.75
Lung consolidation	22 (88)	23 (77)	0.28
Pleural effusions at diagnosis, ultrasound			
Simple (anechoic)	0 (0)	1 (3)	0.36
Complex (septations and/or echogenicity)	25 (100)	29 (97)	
Pleural fluid at diagnosis			
Pus	4 (16)	9 (30)	0.22
pH	7.12 (6.8–7.27)	7.0 (6.99–7.24)	0.34
Positive culture	7 (28)	11 (37)	0.50
Laboratory studies at diagnosis			
Serum C-reactive protein, mg/L	285 (220–374)	270 (196–335)	0.72
Leukocyte count, per µL	14,460 (10,275–19,090)	16,210 (13,200–19,910)	0.18
Positive blood cultures	0 of 22 (0)	2 of 28 (7)	0.20
RAPID score^a^			
Low-risk (0–2)	2 (8)	4 (13)	
Medium-risk (3–4)	15 (60)	23 (77)	0.12
High-risk (5–7)	8 (32)	3 (10)	
Concurrent medications during the study period			
3 to 5-day course of oral azithromycin	9 (36)	11 (37)	0.96
NSAID	16 (64)	25 (83)	0.10
Chest tube bore			
≤ 14F	20 (80)	24 (80)	0.80
16–20F	5 (20)	6 (20)	
Intrapleural fibrinolytics			
Urokinase 100,000 U/d	23 (92)	30 (100)	0.11
Number of doses	1.5 (1–2)	2 (1–3)	0.05
Time from hospital admission to chest tube insertion, days	1 (0–3)	1 (0–3)	0.64
Duration of chest tube drainage, days	1 (0–3)	2 (1–4)	0.05
Days of intravenous amoxicillin-clavulanate	6 (4.5–7)	5 (4–7)	0.42
Length of hospital stay, days	7 (5–8)	6.5 (5–9)	0.86

Data are presented as number (%) or median (quartiles) as appropriate. ^a^The RAPID (renal, age, purulence, infection source, and dietary factors) score consists of 5 clinical factors that can identify patients at risk for increased mortality [10]. COPD, chronic obstructive pulmonary disease; F, French units; mMRC, Modified Medical Research Council; NSAID, non-steroidal anti–inflammatory drugs; VAS, visual analog scale

**Table 2:** Characteristics of the study groups at the time of randomization (day 14).

	2-week treatment group (n=25)	3-week treatment group (n=30)	p-Value
Clinical symptoms			
Chest pain, VAS	1 (0–2)	0 (0–1.3)	0.19
Cough	12 (48)	9 (30)	0.17
Dyspnea, mMRC			
0	7 (28)	14 (47)	
1	9 (36)	13 (43)	
2	7 (28)	2 (7)	0.12
3	2 (8)	1 (3)	
4	0 (0)	0 (0)	
Vital signs			
Temperature, °C	36 (36–36.2)	36 (36–36.2)	0.98
Pulse rate per minute	81 (71–88)	80 (74–93)	0.82
Respiratory rate per minute	17 (15–20)	16 (15–18)	0.60
Systolic blood pressure, mmHg	126 (119–134)	130 (125–141)	0.20
Laboratory studies			
Serum C-reactive protein, mg/L	44 (16–78)	25 (9–67)	0.23
Leukocyte count, per µL	9450 (7100–11,075)	9185 (7313–10,263)	0.86
Effusion’s size at chest radiograph, % of hemithorax	11 (7–15)	10 (7–13)	0.63

Data are presented as number (%) or median (quartiles) as appropriate*.* mMRC, Modified Medical Research Council; VAS, visual analog scale

**Table 3:** Characteristics of the study groups at the end of the intervention period (day 21).

	2-week treatment group (n=25)	3-week treatment group (n=30)	p-Value
Clinical symptoms			
Chest pain, VAS	0 (0–1.5)	0 (0–1)	0.41
Cough	8 (32)	8 (27)	0.66
Dyspnea, mMRC			
0	13 (52)	17 (57)	
1	10 (40)	11 (37)	
2	2 (8)	1 (3)	0.69
3	0 (0)	1 (3)	
4	0 (0)	0 (0)	
Laboratory studies			
Serum C-reactive protein, mg/L	17 (6–42)	9 (6–28)	0.42
Leukocyte count,per µL	6350 (5400–8395)	7540 (6540–9125)	0.12
Effusion’s size at chest radiograph, % of hemithorax	8 (4–10)	5 (3–8)	0.10

Data are presented as number (%) or median (quartiles) as appropriate. mMRC, Modified Medical Research Council; VAS, visual analog scale.

Treatment adherence was optimal, since nearly all 21 envelopes were returned at the end of the intervention period, with the exception of one from a patient in the placebo branch.

### Clinical outcomes

In the intention-to-treat population, clinical success occurred in all 25 patients in the 2-week treatment branch (100%, 95%CI 86–100%) and 29 in the 3-week treatment branch (97%, 95%CI 83–100%, p=0.36). Therefore, the difference between groups for the primary outcome was 3% (95% CI −3 to 9.7%). Since the upper limit of the CI was lower than 10%, the 2-week antibiotic therapy was determined to be non-inferior to the 3-week therapy.

The only patient who failed antibiotic therapy belonged to the 3-week branch and presented with a growing fluid collection at the end of the intervention period, which required a therapeutic thoracentesis along with an additional 2-week course of amoxicillin-clavulanate. During follow-up 3 patients, also from the 3-week branch, died after the original pleuro-pulmonary infection had already been cured: (a) one committed suicide 10 weeks after randomization, (b) another had a fatal traumatic subdural hematoma 6 weeks after randomization, and (c) a third was admitted to the ICU 8 weeks after randomization because of septic shock, with bilateral airspace opacities (without pleural effusion) and isolation of *Pseudomonas aeruginosa* in blood cultures; the initial pleuropulmonary infection was produced by pneumococcus, which was isolated in sputum cultures and also detected in a urinary antigen test. No patient required thoracic surgical intervention.

The last monitoring visit for the evaluation of one of the secondary outcomes could not be performed in four patients: the 3 who had died, and one who had moved to another city, although he was contacted by phone and remained asymptomatic. Among the 51 evaluable subjects, a residual pleural thickening of>10 mm was seen in 13 (59%, 95%CI 36–79%) and 20 (71%, 95%CI 51–87%) patients from the 2- and 3-week branches, respectively (difference −12%, 95%CI −39 to 14%). Consequently, the upper bound of the 95%CI of the difference in percentages of residual pleural thickening between groups did not meet the non-inferiority criterion.

Two patients who had received antibiotics for 3 weeks developed minor adverse events during the blind treatment period: an oral candidiasis that was treated with nystatin swish and swallow, and an antibiotic-associated diarrhea unrelated to *Clostridium difficile* infection, both of which resolved without having to discontinue antibiotics. No significant differences were identified in the rate of adverse events between the 2-week (0%, 95%CI 0–14%) and 3-week (7%, 95%CI 1–22%) groups (difference −7%, 95%CI −16 to 2%).

## Discussion

Randomized controlled trials are considered the gold standard for evaluating the efficacy and safety of therapeutic interventions, but none have so far been conducted to assess the optimal duration of antibiotic therapy in patients with CPPE. The ODAPE trial suggests that a 2-week course of amoxicillin-clavulanate is at least as effective as a 3-week course for those CPPE which meet the entry criteria of the current study.

The 2010 British Thoracic Society guideline for the management of pleural infections recognized that, despite the lack of evidence on this issue, antibiotics are often continued for 3 weeks [12]. More recently, the American Association of Thoracic Surgery recommended a minimum of 2 weeks of antibacterial therapy from the time of drainage and defervescence, based on expert opinion and standard of care [13]. In a recent retrospective review of 91 patients with empyema, of whom 90% underwent a thoracic surgical intervention, the median length of antimicrobial therapy was 27 (IQR 15–31) days [14]. In another series of 140 cases of pleural empyema, antibiotics were administered for a median of 20 days [15].

Unnecessary prolongation of antibiotic therapy may result in increased antibiotic selection pressure that can affect resistance. Likewise, in the ODAPE trial patients who received a longer course of antibiotics had a trend towards developing more adverse effects, which was not statistically significant most likely due to the insufficient sample size. In a multicenter study of 6481 patients hospitalized with pneumonia (73% with CAP), each excess day of treatment was associated with a 5% increase in the odds of antibiotic-associated adverse events reported by patients after discharge (mostly diarrhea, gastrointestinal distress and mucosal candidiasis) [8]. On the other hand, the prevalence of residual pleural thickening at 3 months from diagnosis was about 65%, a figure comparable with that reported in other studies [16]. Nevertheless, this complication has a limited functional impact [17].

It is noteworthy that virtually all patients were initially treated with the instillation of urokinase through a small-bore catheter that remained in place for a short period of time (generally less than 3 days). This management is routine in our practice [18, 19], yet it may differ somewhat from other centers with regard to chest tube size and indwell time, or the use of intrapleural agents. Although the role of intrapleural fibrinolytics alone is still debatable and their combination with DNase is more favored [6, 20], in the ODAPE trial the influence of such a short course of urokinase on the primary end-point was probably negligible.

It should be acknowledged that this trial has limitations. First, the early termination of the trial reduced the statistical power and, owing to the restricted sample size, our findings should be considered preliminary. Nevertheless, even prematurely terminated trials can still yield meaningful results. To be sure, we have been able to demonstrate non-inferiority of 2 vs. 3 weeks of antibiotic therapy using a 10% non-inferiority margin. Admittedly, the study was underpowered to precisely evaluate secondary outcomes (i.e. adverse events, residual pleural thickening). In addition, only 23% of the initially screened patients were eventually enrolled based on the strict entry criteria, which limits the generalizability of the findings. Notably, the results are only valid for a specific subset of patients with community-acquired CPPE, namely those who have a bacterial pathogen non-resistant to amoxicillin-clavulanate, do not have immunocompromise, and are free of chest tubes and obtain clinical stability after 2 weeks of antibiotics. Longer courses of therapy may be indicated in other situations.

In summary, this study is the first to show that for patients with stabilized community-acquired CPPE and no surgical requirement, 3 weeks of antimicrobial treatment appears to have no clinical advantage over 2 weeks of antibiotics. Given the premature termination of the trial, firm conclusions await further confirmatory studies.
